# ﻿*Epipyxisfenheensis* sp. nov., a new species of the genus *Epipyxis* (Chrysophyceae)

**DOI:** 10.3897/phytokeys.260.154552

**Published:** 2025-07-28

**Authors:** Jun-Xue Hao, Ya-Lu An, Fang-Ru Nan, Jun-Ping Lv, Qi Liu, Xu-Dong Liu, Shu-Lian Xie, Jia Feng

**Affiliations:** 1 School of Life Science, Shanxi Key Laboratory for Research and Development of Regional Plants, Shanxi University, Taiyuan 030006, China Shanxi University Taiyuan China

**Keywords:** *Epipyxis*, evolution, molecular phylogeny, species diversity, taxonomy

## Abstract

The genus *Epipyxis*, belonging to the family Dinobryaceae, has been documented to have only sporadic occurrences in freshwater habitats. However, the species diversity of this genus remains largely unexplored due to the scarcity of available molecular sequences. This limitation has significantly hindered a comprehensive understanding of both the species diversity and evolutionary relationships of the genus *Epipyxis*. In this study, a new species *Epipyxisfenheensis***sp. nov.** was described from Shanxi Province, China, based on detailed morphological observations and phylogenetic analyses. This species was characterized by a tube-like lorica, a spindle protoplast, two heterokont flagella, and oval or elliptic scales. In addition, phylogenetic analysis based on multi-genes (SSU, LSU, and *rbc*L) indicated that strain SX231009 was closely related to *E.pulchra*. Given its distinct morphological characteristics and independent phylogenetic position, we propose the designation of this strain as a new species, *E.fenheensis***sp. nov.** The results of this study significantly expand the known diversity of the genus *Epipyxis* and provide valuable insights into the regional biodiversity and evolutionary history of freshwater chrysophytes.

## ﻿Introduction

*Epipyxis*, a rare member of Dinobryaceae, was formally established in 1838, with *Epipyxisutriculus* designated as its type species (Ehrenberg 1838). Initially, *E.utriculus* was classified within the genus *Dinobryon* (Wille 1882; Klebs 1893). Stein (1878) was the first to place flagellates within the family Chrysomonadaceae. The classification of *Epipyxis* evolved with the recognition of orders, ultimately leading to its inclusion in the order Ochromonadales (Pascher 1910, 1912). In 1914, the class Chrysophyceae was formally established, and flagellates were reorganized within Chrysomonadales (Pascher 1914). By the 20^th^ century, *Epipyxis* had firmly established its status as a distinct genus (Krieger 1930; Ahlstrom 1937; Smith 1950; Fott 1959). Bourrelly (1965, 1968, 1981) proposed influential classification schemes, dividing Chrysophyceae into three subclasses: Acontochrysophycidae, Heterochrysophycidae, and Isochrysophycidae. He recognized two orders within the Heterochrysophycidae and placed taxa with two unequal flagella under Ochromonadales based on flagellar length and number. Starmach’s classification system followed Bourrelly’s framework, maintaining choanoflagellates as members of the Chrysophyceae (Starmach 1985). In 1999, Andersen conducted a groundbreaking phylogenetic analysis, confirming the taxonomic position of *Epipyxis* within Chrysophyceae (Andersen et al. 1999). More recently, the classification systems proposed by Kristiansen et al. (2001) and Guiry and Guiry (2024) have continued to affirm the taxonomic status of *Epipyxis* as a member of the family Dinobryaceae.

The genus *Epipyxis* is particularly challenging to detect and study due to its extreme fragility and transparency (Hilliard 1959). The unique organism thrives in specialized habitats, predominantly within sphagnum-rich bogs and freshwater ponds in the temperate, subarctic, and arctic regions of Europe and North America, with notable abundance in Canada and Alaska (Whelden 1947; Hilliard 1959). *Epipyxis* exhibits an epiphytic lifestyle and displays a dual mode of existence, thriving both as a colony (Thompson 1959) and as single cells (Whelden 1947; Smith 1920). Its fundamental structure is a loricate monad, with the lorica exhibiting various shapes, including tubular, cylindrical, and subcylindrical forms. The surface of loricae may also be adorned with organic scales (Hilliard and Asmund 1963). Morphological variation serves as a key criterion for species identification of this genus. Each cell of *Epipyxis* species possesses two unequal flagella. The shorter flagellum, though often overlooked, plays a pivotal role in phagocytosis—the process of ingesting food particles (Andersen and Wetherbee 1992; Wetherbee and Andersen 1992). In some cases, phagocytosis is facilitated by the assistance of nearby root-like structures, highlighting the remarkable adaptability and survival strategies of the genus *Epipyxis* (Wujek 1968; Preisig and Hibberd 1982a, 1982b; Preisig and Hibberd 1983; Andersen 1990).

To date, a total of 33 *Epipyxis* species have been accepted in the AlgaeBase (Guiry and Guiry 2024, accessed on 17 Mar. 2025). Among these, only two species, *E.aurea* and *E.pulchra*, have been supported by molecular evidence (Daugbjerg and Andersen 1997; Andersen et al. 1999; Andersen 2007). However, molecular phylogenetic studies on this genus remain scarce, with only SSU and *rbc*L sequences available for two species. Across the vast geographical region of China, only five species of the genus *Epipyxis* have been documented (Xie 2018). In 2019, Pang et al. reported four species records, including *E.epiplanctica*, *E.proteus*, *E.deformans*, and E.utriculusvar.pusilla, based solely on detailed morphological observations (Pang et al. 2019). The lack of molecular evidence underscores the urgent need for further exploration of the species diversity of freshwater chrysophyta in China.

In this study, we propose a new species, *Epipyxisfenheensis* sp. nov., based on comprehensive morphological characterization and molecular evidence. The primary objectives of this study were: (1) to describe morphological characterizations of the new *Epipyxis* species, (2) to supplement the molecular data of the genus *Epipyxis*, (3) to elucidate the relationships between *Epipyxis* species and other related taxa, (4) to contribute to a deeper understanding of the evolutionary history of chrysophytes.

## ﻿Methods

### ﻿Collection and culture

**Figure 1.**
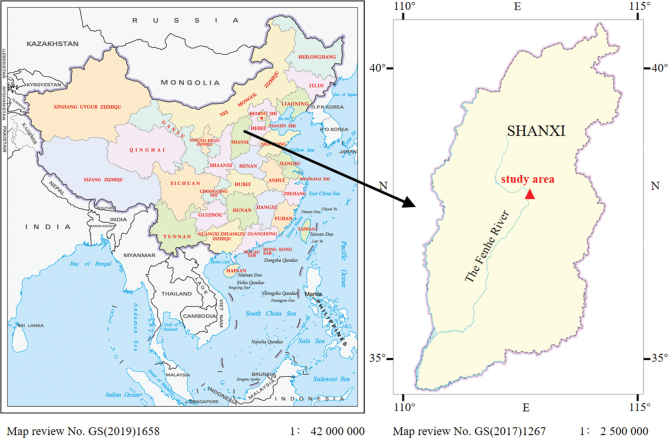
Sampling location of *Epipyxisfenheensis* sp. nov. in China.

The sample was collected in October 2023 from the Fenhe River in Shanxi Province, China (37°50.12'N, 112°32.13'E) (Fig. 1). Individuals were gathered using a 20-μm mesh plankton net and immediately transported to the laboratory. Individual flagellate cells were isolated under an inverted microscope (Motic AE31) using Pasteur pipettes. The isolated cells were then cultured in 24-well plates, with each well containing 2.0 mL of DY-IV or WC medium, and the pH was adjusted to maintain a range of 6.8–8.0. Cultures were maintained at 12–16 °C under a 12-h/12-h light/dark cycle. Voucher specimen was preserved in 4% formaldehyde and deposited in the Herbarium of Shanxi University (SXU), Shanxi University, Taiyuan, Shanxi Province, China.

### ﻿Morphological observation

According to the morphological characteristics described in the previous study (Pang et al. 2019), the structures were observed and photographed under an Olympus BX-51 microscope equipped with a charge-coupled device (DP72; Olympus, Tokyo, Japan). The scales were observed using Jensen staining (Hilliard and Asmund 1963). 10 mL of the culture was concentrated by centrifugation at 3000 rpm for 5 minutes. A small aliquot of the concentrated sample was evenly spread onto a glass slide, air-dried, and rapidly fixed by passing it through a flame 2–3 times. The staining protocol initiated with 1–2 minutes of crystal violet treatment, followed by gentle distilled water rinsing to remove excess dye. Subsequently, the sample was treated with Lugol’s iodine solution as a mordant for approximately 1 minute, after which they were rinsed slowly with distilled water. Decolorization was performed by applying 95% ethanol, gently agitating the slide for 10–20 seconds, and immediately rinsing with distilled water to halt the process. Finally, the sample was counterstained with safranin solution for 0.5–1 minute and rinsed again with distilled water. The stained scale structures were then examined under an optical microscope. The loricae were observed by methylene blue staining (Hilliard 1964). Air-dried samples were stained with methylene blue for 3–5 minutes, followed by distilled water rinsing prior to microscopic observation. The formula of dyeing reagents is shown in Table 1.

**Table 1.** Dyeing reagent formula.

Dyeing reagent	Formula
Primary stain	Crystal Violet: 2 g
	95% ethanol: 20 mL
	Distilled water: 80 mL
Distilled water	I_2_: 1 g
	KI:2 g
	Distilled water: 300 mL
Counter stain	Safranin: 0.5 g
	Distilled water: 100 mL
Methylene blue aqueous solution	Methylene blue: 0.1 g
	Distilled water: 100 mL

### ﻿DNA extraction, amplification and sequencing

Total DNA was extracted using the EasyPure Plant Genomic DNA Kit (TransGen Biotech, China) and stored at -80 °C for subsequent analysis. Polymerase chain reaction (PCR) amplification of the small subunit (SSU), large subunit (LSU), and ribulose-1,5-bisphosphate carboxylase/oxygenase large subunit (*rbc*L) genes was performed in a 50 μL reaction mixture containing 37.75 μL of ddH_2_O, 5 μL of 10×buffer, 4 μL of 2.5 mM dNTPs, 1 μL of each forward and reverse primer (10 μmol/L), 1 μL of DNA template, and 0.25 μL of Taq DNA polymerase sourced from Sangon Biotech Co., Ltd., China. PCR amplification was performed using a MyCycler thermal cycler (Bio-Rad, Hercules, CA, USA) under the following conditions: initial denaturation at 94 °C for 5 minutes; 35 cycles of denaturation at 94 °C for 30–60 seconds, annealing at 44–51 °C for 30–60 seconds, and extension at 72 °C for 2 minutes; followed by a final extension at 72 °C for 7–10 minutes. Primer sequences for amplifying the target genes were adopted from Katana et al. (2001), Jo et al. (2011), Pusztai and Škaloud (2022), and Jeong et al. (2021), as detailed in Table 2. The PCR products were separated by agarose gel electrophoresis, and those exhibiting clear bands were sent to BGI Tech Corporation in Beijing, China for sequencing using an ABI 3730XL sequencer. The obtained sequences were deposited in GenBank under the following accession numbers: PQ364874, PQ364873, PQ368406, and PQ374158.

**Table 2.** Primers used in this study.

Primer name	Sequence (5’–3’)	Target gene	Reference
18SF	AACCTGGTTGATCCTGCCAGT	SSU	(Katana et al. 2001)
18SR	TGATCCTTCTGCAGGTTCACCTACG		
28S_25F	ACCCGCTGAATTTAAGCATATA	LSU	(Jo et al. 2011)
28S_861R	GTTCGATTAGTCTTTCGCCCCT		
28S_736F	CCCGAAAGATGGTGAACTC		
28S_1440R	TGCTGTTCACATGGAACCTTTC		
28S_1228F	CCTGAAAATGGATGGCGC		
28S_2160R	CCGCGCTTGGTTGAATTC		
28S_2038F	GACAAGGGGAATCCGACT		
28S_2812R	GATAGGAAGAGCCGACATCGAA		
Chryso_*rbc*L_F4	TGGACDGAYTTATTAACDGC	*rbc*L	(Pusztai and Škaloud 2019)
Chryso_*rbc*L_R7	CCWCCACCRAAYTGTARWA		
ITS_DF	CGCACCTACCGATTGAAT	ITS	(Jeong et al. 2021)
ITS_DR	CCTCCGCCTAGTTATATGCTTA		

### ﻿Sequence alignment and phylogenetic analysis

For phylogenetic analyses, sequence data of *Epipyxis* species and other related taxa were downloaded from Genbank to construct phylogenetic trees based on concatenated SSU, LSU, and *rbc*L sequences, with *Synochromagrande* and *Nannochloropsislimnetica* designated as outgroup taxa. Sequence alignment was performed with MAFFT v.7 (Katoh et al. 2019) and manually refined. Pairwise distances of sequence variation were calculated using MEGA v.5 (Tamura et al. 2011). The sequences of the three genes were concatenated following the methodology described by Zhang et al. (2020). The appropriate models for the sequence evolution were determined using the software PartitionFinder v.2 (Lanfear et al. 2017). For both Bayesian inference (BI) and Maximum Likelihood (ML) analyses, the best model of subsets (1)(2)(3) was identified as GTR+I+G, with the following substitution rates: A–C = 0.8710, A–G = 3.1453, A–T = 1.2999, C–G = 0.7034, and C–T = 5.2671. Bayesian inference (BI) was performed using Mrbayes v.3.2.6 (Ronquist et al. 2012), with the analysis run for 5,000,000 generations. Additionally, Maximum Likelihood (ML) trees were constructed using IQ-TREE v.1.6.12, employing 5000 ultrafast bootstrap replicates and a Shimodaira-Hasegawa-like approximate likelihood-ratio test for validation (Guindon et al. 2010; Minh et al. 2013; Nguyen et al. 2015). The resulting phylogenetic trees were visualized and edited using FIGTREE v.1.4.2 (http://tree.bio.ed.ac.uk/software/figtree/). Final graphical optimizations of the trees were performed using Adobe Illustrator (Adobe System, San Jose, CA, USA).

### ﻿Molecular clock analyses

Divergence time estimation was conducted using a relaxed clock model implemented in BEAST v.2.6.6 (Bouckaert et al. 2014). Since the phylogenetic trees based on single-gene and multi-gene datasets exhibited similar topology, a Bayesian Inference was used to construct molecular clock phylogeny based on concatenated SSU, LSU, and *rbc*L sequences, for taxa lacking LSU/*rbc*L data, only the existing sequences were used. The uncorrelated lognormal model was used to estimate variation rates, with fossil calibrations incorporated as probabilistic priors. Fossil records of *Mallomonas* from the Giraffe Pipe locality were used to calibrate the stem nodes for the divergence between *Mallomonasdenticulata* and M.striatavar.serrata, as well as between *M.elevata* and *M.foveata* (Jo et al. 2013; Siver et al. 2015). Both calibrations were based on a time offset of 38 million years ago (Ma), with a mean value of 0.5 Ma and a standard deviation of 1.0, providing a minimal age approximation (Creaser et al. 2004; Doria et al. 2011; Siver et al. 2015). A generalized time-reversible (GTR) model with gamma-distributed site heterogeneity was selected, and a Yule tree prior was applied for the estimation. The analysis was run for 30 million generations, with sampling conducted every 1,000 generations. The convergence of parameter estimates and burn-in determination was assessed using TRACER v.1.7.1 (Rambaut et al. 2018). The initial 3000 trees (representing 30 million generations) were discarded as burn-in, and the remaining 27,000 trees were used to generate the final chronogram. Node ages were estimated with 95% posterior probabilities (PP). The resulting phylogenetic trees were visualized using FIGTREE v.1.4.2 (http://tree.bio.ed.ac.uk/software/figtree/) and further refined for presentation using Adobe Illustrator CS5 (Adobe System, San Jose, CA, USA).

### ﻿ITS2 secondary structures

The ITS2 sequences of *Mallomonas* and *Synura* were retrieved from GenBank and aligned with the newly obtained sequence of *Epipyxis* from this study using MAFFT v. 7 (Katoh et al. 2019). The ITS2 secondary structure of *Epipyxis* specimen was predicted using the mfold program (Zuker 2003) and visualized with VARNA (Darty et al. 2009).

## ﻿Results

### ﻿Taxonomic treatment

#### ﻿ Epipyxis fenheensis

Taxon classificationPlantaeOchromonadalesDinobryaceae

J.Hao, J.Feng, S.Xie sp. nov.

2B6E8BCF-9424-5526-B1C1-8146A3F770FC

Figs 2 , 3 , 4

##### Type.

China • Shanxi Province, Taiyuan City, the Fenhe River; 37°50.12'N, 112°32.13'E; 9 Oct. 2023; Yalu An and Junxue Hao leg.; phytoplankton; ***Holotype***: SXU-SX231009; GenBank: PQ364874, PQ364873, PQ368406 and PQ374158.

**Figure 2.**
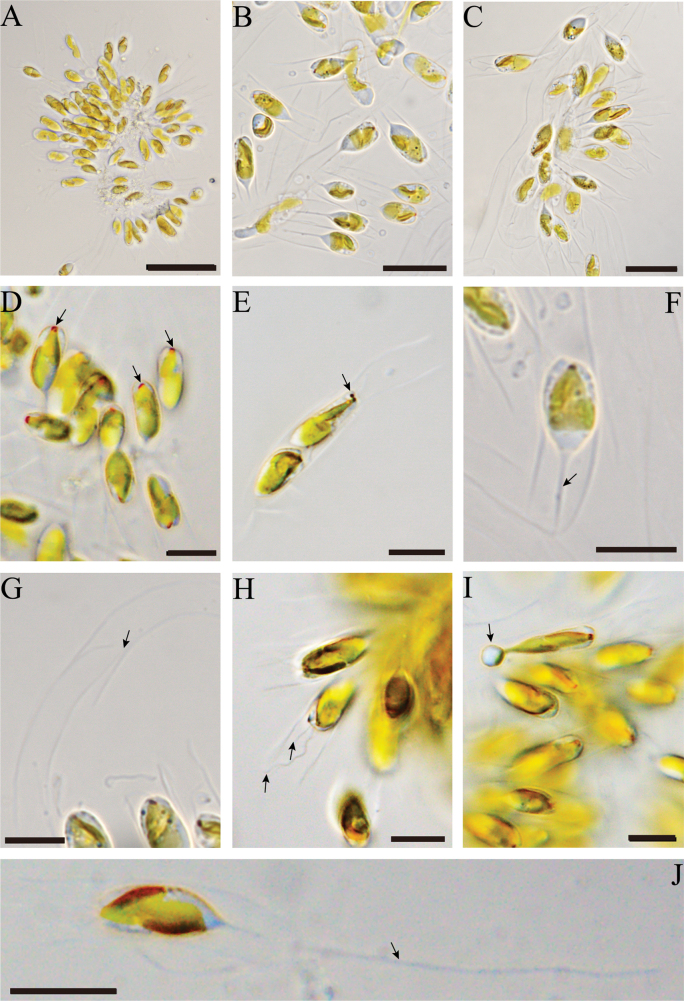
Morphological structures of *Epipyxisfenheensis* sp. nov. observed by light microscope (LM) **A.** Morphology of colony; **B, C.** Scattered protoplasts and loricae; **D.** Protoplast with a red stigma, as indicated by arrows; **E.** Protoplast with two red stigma, as indicated by an arrow; **F.** Protoplast spindle, the posterior end of the protoplast is obviously pointed, tapering at the base to a delicate stalk, as indicated by an arrow; **G.** Long, tube-like lorica, the junction of loricae is indicated with an arrow; **H.** Two unequal flagella as indicated by an arrow; **I.** Reproducing cell; **J.** The posterior end of lorica with a long thick root, as indicated by an arrow. Scale bars: 50 μm (**A**); 20 μm (**B, C**); 10 μm (**D–J**).

##### Description.

Cells are small, loricate monads with two heterokont flagella, and encased within a lorica. The lorica is tubular, measuring 28.3–43.2 µm in length and 4.78–10.63 µm in width, with subparallel lateral margins. The upper opening of the lorica exhibits a slight expansion or remains parallel. The posterior end of the lorica is slightly constricted and attached to a long thick root, the same length as lorica. The lorica surface is smooth, adorned with oval or elliptical scales, arranged in an obliquely parallel, imbricate pattern. The protoplast is spindle-shaped, with dimensions ranging from 7.27–26.7 μm long and 3.6–10.56 μm wide. It possesses two unequal flagella and a red stigma. The longer flagellum matches the length of the protoplast, whereas the shorter flagellum is approximately one-third of its length. The posterior end of the protoplast is acutely pointed, tapering at the base into a delicate stalk, which does not form a stipe. The stalk measures 7.07–12 μm in length, slightly shorter than the protoplast. Cells are epiphytic and tend to form clumpy colonies. Younger individuals are typically attached to the inner surface of the maternal lorica.

**Figure 3.**
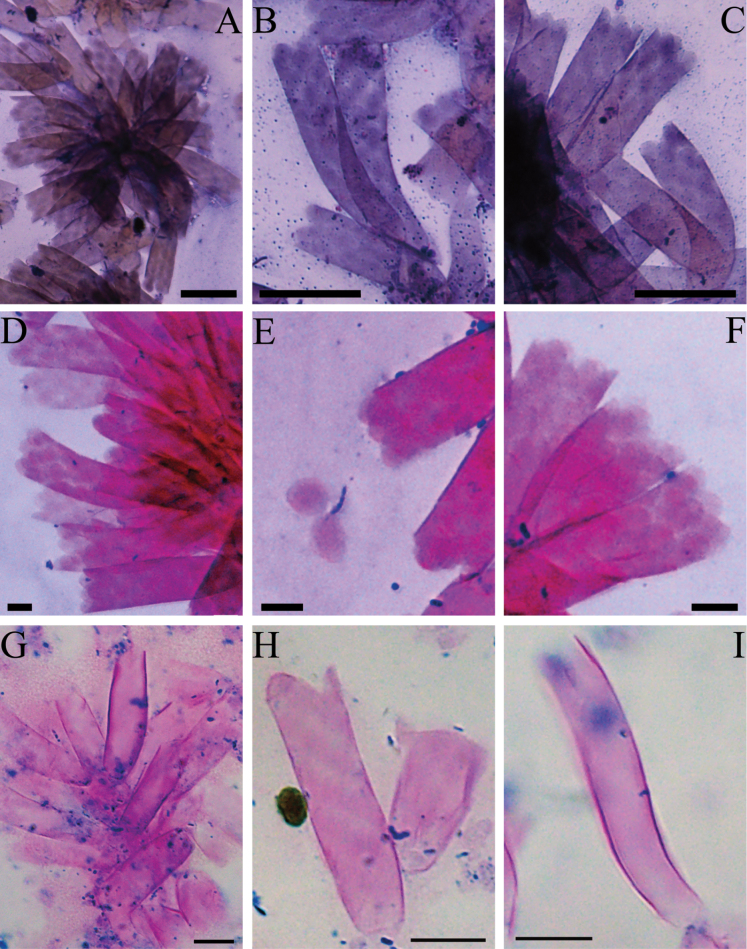
Light micrographs of *Epipyxisfenheensis* sp. nov. after staining **A.** Cell colony; **B–D, F.** Loricae with scales; **E.** Falling oval and elliptical scales; **G.** Cell colony; **H.** Loricae with scales arranged diagonally and parallel; **I.** Smooth lorica surfaces. Scale bars: 20 µm (**A–C**); 10 µm (**G–I**); 5 µm (**D–F**).

##### Etymology.

The species epithet refers to the type locality (the Fenhe River, Taiyuan City, Shanxi Province, China).

##### Authentic strain.

SXU-SX231009. Deposited in Herbarium of Shanxi University (SXU), Shanxi University, Taiyuan, Shanxi Province, China.

### ﻿Life history observation

**Figure 4.**
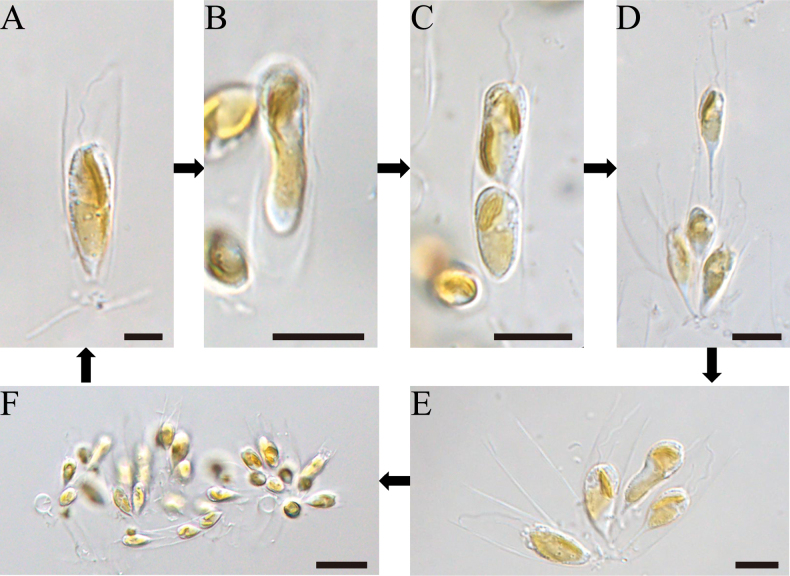
Life history of *Epipyxisfenheensis* sp. nov. **A.** A single cell; **B.** Single cell division; **C.** Division of a single cell into two cells; **D.** Separation of two protoplasts; **E.** Multiple cells; **F.** Colony. Scale bars: 5 µm (**A–D**); 10 µm (**E**); 20 µm (**F**).

The genus *Epipyxis* reproduces vegetatively through longitudinal cell division. Observations of the life cycle of *Epipyxisfenheensis* sp. nov. (Fig. 4) reveal that the complete life cycle spans approximately 7 days. On the first day (Fig. 4A), the cell existed as a single entity in its initial stage, gradually enlarging prior to division. During this phase, the cell displayed a relatively simple structure, with no evident signs of division. By the second day (Fig. 4B), the cell initiated division. At this stage, the internal cellular structure underwent significant reorganization, including nuclear and cytoplasmic redistribution. On the third day (Fig. 4C), cell division was completed, yielding two distinct cells. At this point, the protoplasts of the two cells remained closely associated but showed clear signs of separation. Each cell possessed a full complement of organelles and genetic material. By the fourth day (Fig. 4D), the stalks of the daughter cells elongated and became more slender, fully separating from the mother cell. Each daughter cell entered a new lorica, establishing itself as an independent entity. These cells commenced autonomous growth and development, preparing for subsequent rounds of division. On the fifth day (Fig. 4E), after several days of growth and division, a cluster of multiple cells was formed. By the seventh day (Fig. 4F), the cell population had expanded significantly, forming an extensive colonial structure.

### ﻿Phylogenetic analysis

**Figure 5.**
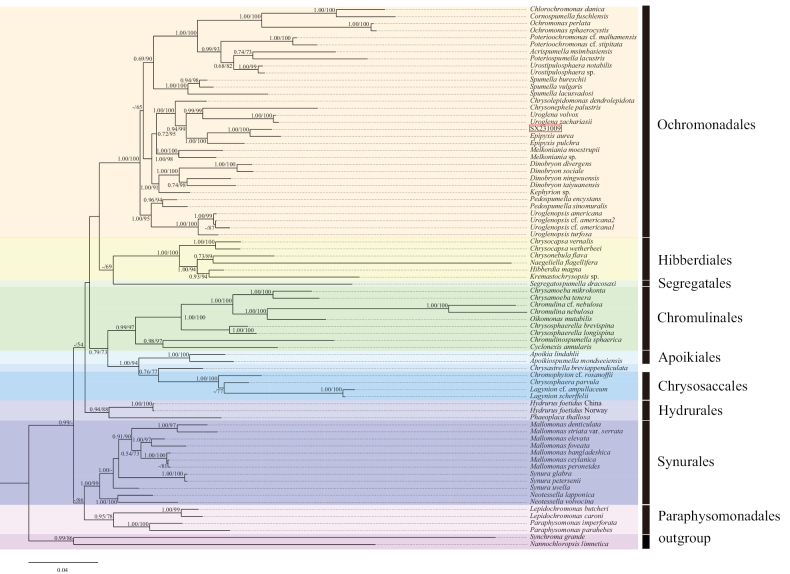
The phylogenetic tree constructed based on SSU sequence (Bayesian inference/maximum likelihood method), the Bayesian tree was selected here for display, and the values of the branch nodes correspond to the Bayesian posterior probabilities (left) and the Maximum likelihood bootstraptree support values (right). Node support values below 50% are indicated by “-”, and the sample of the genus *Epipyxis* in this study is indicated in red boxes.

**Figure 6.**
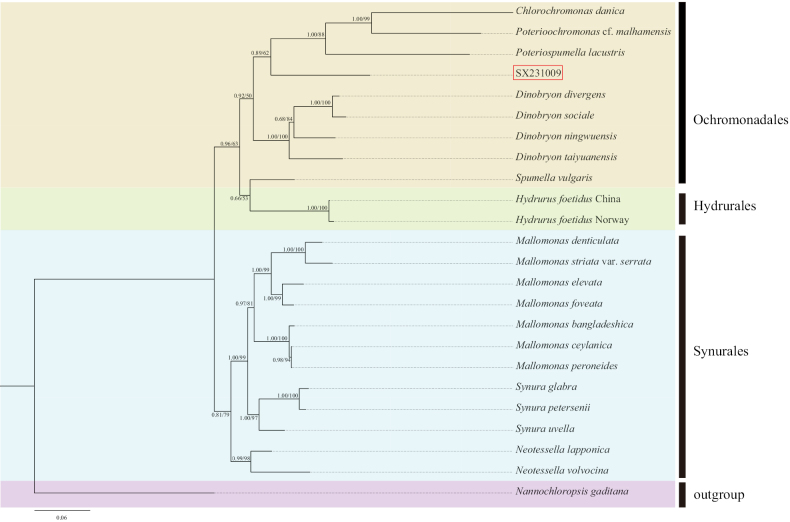
The phylogenetic tree constructed based on LSU sequence (Bayesian inference/maximum likelihood method), the Bayesian tree was selected here for display, and the values of the branch nodes correspond to the Bayesian posterior probabilities (left) and the Maximum likelihood bootstraptree support values (right). Node support values below 50% are indicated by “-”, and the sample of the genus *Epipyxis* in this study is indicated in red boxes.

**Figure 7.**
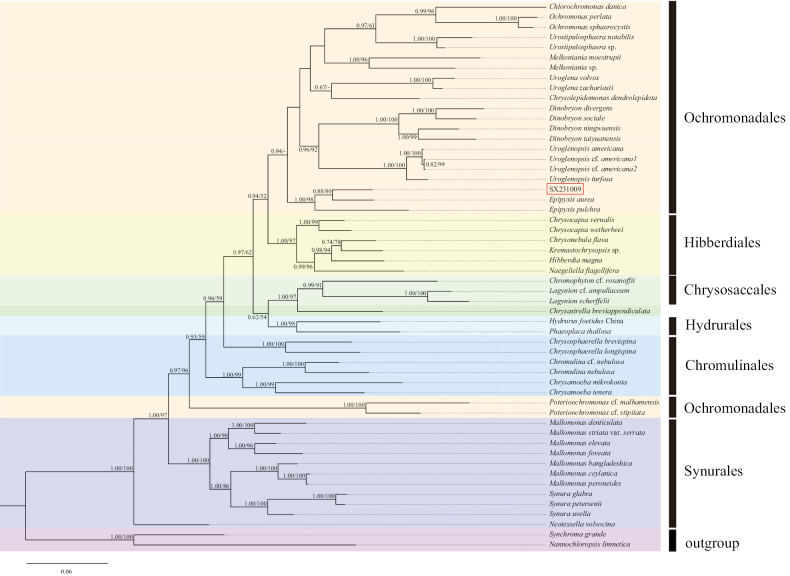
The phylogenetic tree constructed based on *rbc*L sequence (Bayesian inference/maximum likelihood method), the Bayesian tree was selected here for display, and the values of the branch nodes correspond to the Bayesian posterior probabilities (left) and the Maximum likelihood bootstraptree support values (right). Node support values below 50% are indicated by “-”, and the sample of the genus *Epipyxis* in this study is indicated in red boxes.

**Figure 8.**
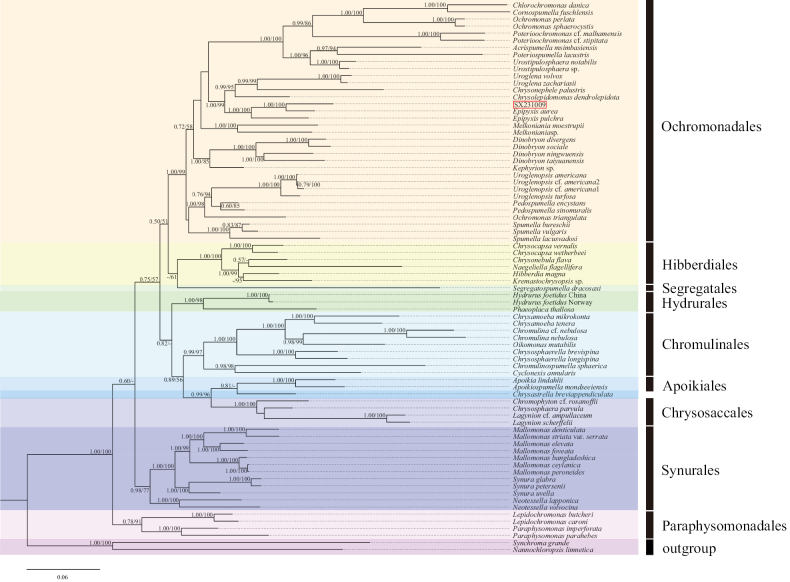
The phylogenetic tree constructed based on concatenated SSU, LSU, and *rbc*L sequences (Bayesian inference/maximum likelihood method), the Bayesian tree was selected here for display, and the values of the branch nodes correspond to the Bayesian posterior probabilities (left) and the Maximum likelihood bootstraptree support values (right). Node support values below 50% are indicated by “-”, and the sample of the genus *Epipyxis* in this study is indicated in red boxes.

We conducted a phylogenetic analysis using 74 taxa, comprising 72 chrysophytes and 2 outgroups (Suppl. material 1). Pairwise genetic distances based on concatenated SSU, LSU, and *rbc*L genes are respectively listed in Suppl. materials 2–4. Both Bayesian Inference (BI) and Maximum Likelihood (ML) methods yielded largely congruent topologies with high support values. Therefore, we present only the BI trees (with full support values) in Figs 5–8.

The SSU dataset comprised 77 sequences, including 3 sequences from the genus *Epipyxis*, 72 sequences from other genera within Chrysophyceae, and 2 outgroup sequences. Following alignment and trimming, the SSU sequences comprised 1759 bp, of which 1145 bp (65.09%) were conserved sites, 577 bp (32.80%) were variable sites, and 399 bp (22.68%) were parsimony-informative sites. In the phylogenetic tree constructed based on SSU sequences (Fig. 5), the genus *Epipyxis* formed a well-supported monophyletic clade. The strain SX231009 showed a close relationship with *Epipyxisaurea* and *E.pulchra* (1.00/100). Specifically, SX231009 and *E.aurea* clustered together into a distinct subclade, supported by full values (1.00/100). The SSU sequences of the strain SX231009 and *E.aurea* differed by 12 nucleotides (out of 1732 bp).

The LSU dataset comprised 24 sequences, including 1 sequence from the genus *Epipyxis*, 22 sequences from other genera within Chrysophyceae, and 1 outgroup sequence. Following alignment and trimming, the LSU sequences comprised 2906 bp, of which 1936 bp (66.62%) were conserved sites, 862 bp (29.66%) were variable sites, and 600 bp (20.65%) were parsimony-informative sites. In the phylogenetic tree constructed based on LSU sequences (Fig. 6), the strain SX231009 is closely related to *Poteriospumella*, *Poterioochromonas*, and *Chlorochromonas* (0.89/62).

The *rbc*L dataset comprised 53 sequences, including 3 sequences from the genus *Epipyxis*, 49 sequences from other genera within Chrysophyceae, and 2 outgroup sequences. Following alignment and trimming, the *rbc*L sequences comprised 968 bp, of which 505 bp (52.17%) were conserved sites, 463 bp (47.83%) were variable sites, and 416 bp (42.98%) were parsimony-informative sites. In the phylogenetic tree constructed based on *rbc*L sequences (Fig. 7), the genus *Epipyxis* formed a well-supported monophyletic clade. The strain SX231009 showed a close relationship with *Epipyxisaurea* and *E.pulchra* (1.00/100). Specifically, SX231009 and *E.aurea* clustered together into a distinct subclade, supported by full values (1.00/100). The *rbc*L sequences of the strain SX231009 and *E.aurea* differed by 41 nucleotides (out of 948 bp).

The concatenated sequence dataset comprised 74 taxa, including 74 SSU sequences, 21 LSU sequences, and 51 *rbc*L sequences. The combined dataset had a total length of 6202 bp, of which 2044 bp (32.96%) were variable sites, 3922 bp (63.24%) were conserved sites, and 1544 bp (24.90%) were parsimony-informative sites. The average nucleotide composition was 28.5% thymine (T), 18.2% cytosine (C), 28.3% adenine (A), and 25.0% guanine (G). In the phylogenetic tree constructed based on concatenated sequences (Fig. 8), within the Order Ochromonadales, the genera *Poterioochromonas*, *Urostipulosphaera*, *Uroglena*, *Epipyxis*, *Melkoniana*, *Dinobryon*, *Uroglenopsis*, and *Spumella* were monophyletic, whereas the genus *Ochromonas* was polyphyletic. The genus *Epipyxis* exhibited a close relationship with the genera *Chrysolepidomonas*, *Chrysonephele*, and *Uroglena*, supported by high values (1.00/99). The strain SX231009 is closely related to *Epipyxisaurea* and *E.pulchra* by full values (1.00/100). Phylogenetic analysis revealed that the strain SX231009 and *E.aurea* form an independent lineage within the well-supported Order Ochromonadales. Within the Order Hibberdiales, the genus *Chrysocapsa* (including *Chrysocapsavernalis* and *C.wetherbeei*) was monophyletic. In the Order Chromulinales, the genera *Chrysamoeba* (including *Chrysamoebamikrokonta* and *C.tenera*), *Chromulina*, *Chrysosphaerella* (including *Chrysosphaerellabrevispina* and *C.longispina*) were also monophyletic. Within the Order Chrysosaccales, the genus *Lagynion* was monophyletic. Within the Order Synurales, the genus *Mallomonas* was closely related to the genus *Synura*, with the genus *Neotessella* positioned at the base of this Order. Within the Order Paraphysomonadales, the genera *Lepidochromonas* (including *Lepidochromonasbutcheri* and *L.caroni*) and *Paraphysomonas* (including *Paraphysomonasimperforata* and *P.parahebes*) were monophyletic.

### ﻿Molecular clock analyses

**Figure 9.**
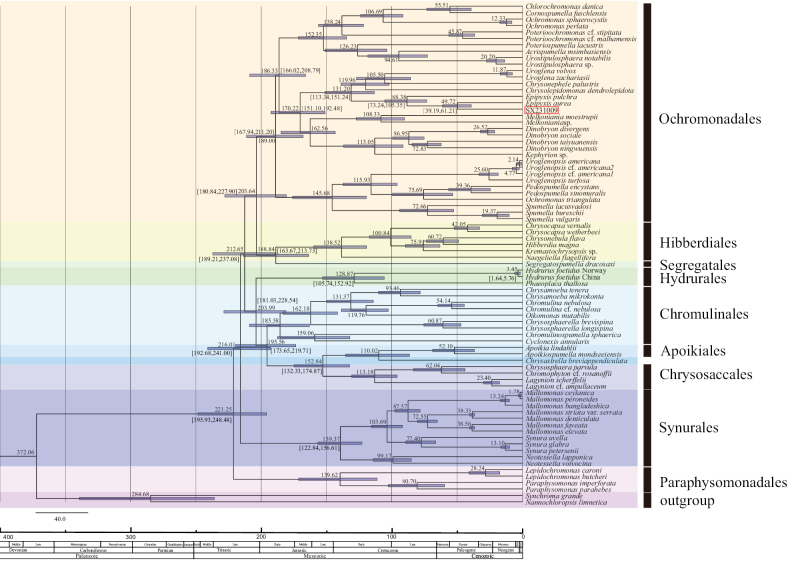
Divergence time estimation phylogeny based on concatenated SSU, LSU and *rbc*L sequences, the number of branch nodes represents ages. Blue horizontal bars indicate 95% height posterior density. The strain of the genus *Epipyxis* in this study is indicated in red boxes. The geological timescale is measured in million years ago.

We inferred the BEAST trees based on concatenated SSU, LSU, and *rbc*L genes to estimate the origin of species within Chrysophyceae (Fig. 9). Our time-calibrated phylogenetic analysis primarily used fossil calibrations from the Giraffe Pipe locality. The phylogenetic tree showed that the divergence between strain SX231009 and *Epipyxisaurea* occurred 49.75 million years ago (Ma). *E.pulchra* diverged from *E.aurea* and strain SX231009 between 73.24 Ma and 105.35 Ma, with the genus *Epipyxis* originating approximately in the Early Cretaceous (131.20 Ma). Within Order Ochromonadales, the genus *Chlorochromonas* diverged from the genus *Cornospumella* approximately at 55.51 Ma. The genus *Poterioochromonas* originated around 138.24 Ma. The genus *Urostipulosphaera* diverged from *Acrispumella* likely during the Cretaceous (94.61 Ma). The divergence of *Uroglena* from *Chrysonephele* occurred at 105.50 Ma, and the genus *Melkoniana* diverged from *Dinobryon* and *Kephyrion* during the Jurassic period. The genus *Uroglenopsis* likely originated in the Early Cretaceous, and the genus *Spumella* originated around 145.68 Ma. Within Order Hibberdiales, the genus *Chrysocapsa* diverged approximately at 100.84 Ma. The Order Segregatales originated during the Early Jurassic, while the Order Hydrurales emerged during the Late Triassic. Within the Order Chromulinales, the genus *Chrysamoeba* diverged from *Chromulina* and *Oikomonas* approximately at 131.37 Ma, and the genus *Chrysosphaerella* originated at 162.18 Ma, likely during the Jurassic. The genera *Chromulinospumella* and *Cyclonexis* diverged approximately at 159.06 Ma. The Order Apoikiida originated during the Early Cretaceous. Within the Order Chrysosaccales, the genus *Lagynion* diverged from *Chrysosphaera* and *Chromophyton* at 113.18 Ma (Early Cretaceous). The Order Synurales originated during the Late Triassic, with *Neotessella* diverging from *Mallomonas* and *Synura* around 139.37 Ma. Within the Order Paraphysomonadales, the genera *Clathromonas* and *Paraphysomonas* diverged at 139.62 Ma, most likely in the Early Cretaceous.

### ﻿ITS2 secondary structures

**Figure 10.**
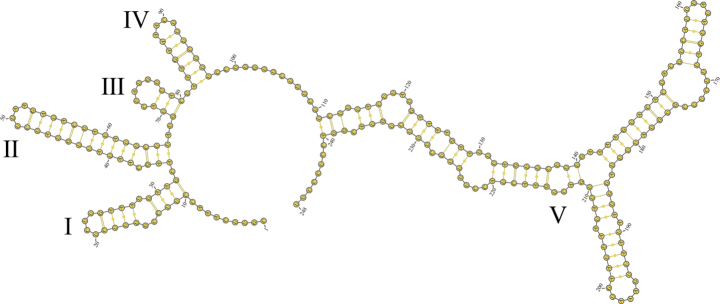
ITS2 secondary structure of *Epipyxisfenheensis* sp. nov. Base numbering is indicated every 10 bases.

The ITS region (ITS1-5.8S-ITS2) of *Epipyxisfenheensis* sp. nov. was successfully sequenced, revealing an ITS2 segment of 248 bp. The predicted ITS2 secondary structure adopted a conserved “ring-pin” conformation (Fig. 10). The ITS2 secondary structure of *E.fenheensis* sp. nov. was characterized by five extended stems primarily stabilized by Watson-Crick base-pairing interactions. Structural analysis showed distinct features. Paired regions (56.29%) predominated over unpaired regions. The helix regions were more extensive than the loop regions. Bulge regions accounted for 13.71% of the total sequence length. The base numbers of A, U, C, and G and pairing composition of ITS2 are detailed in Table 3. Base composition analysis revealed a distinct hierarchy (U > A > C > G), with AU, GC, and GU base pairs respectively predominating at 68.00%, 21.33%, and 10.67% of total nucleotides. Comparative structural analysis showed differential base distribution across helices. Helix IV displayed balanced purine/pyrimidine content (1:1 ratio). The remaining four helices exhibited pyrimidine dominance.

**Table 3.** Base and pairing composition of ITS2.

	A (%)	U (%)	C (%)	G (%)	Purines/pyrimidines	CG(GC) (%)	AU(UA) (%)	GU(UG) (%)
Total	28.63	42.34	16.94	12.10	0.69	21.33	68.00	10.67
Paired region	31.31	42.06	13.56	13.08	0.80	21.33	68.00	10.67
Helix I	21.74	43.48	21.74	13.04	0.53	37.50	62.50	0.00
Helix II	36.36	39.40	12.12	12.12	0.94	21.43	71.43	7.14
Helix III	25.00	41.67	16.67	16.67	0.71	33.33	66.67	0.00
Helix IV	43.75	37.50	12.50	6.25	1.00	16.67	83.33	0.00
Helix V	30.77	43.08	12.31	13.85	0.81	18.18	65.91	15.91

## ﻿Discussion

The genus *Epipyxis* exhibits an epiphytic lifestyle, with individuals existing in both colonial and solitary forms (Whelden 1947; Thompson 1959). Hilliard and Asmund (1963) reported several new species of this genus, including *Epipyxiscondensata*, *E.gracilis*, *E.kenaiensis*, *E.polymorpha*, and *E.ramosa*. More recently, Pang et al. (2019) documented additional species records of *E.epiplanctica*, *E.proteus*, *E.deformans*, and E.utriculusvar.pusilla. Despite these contributions, taxonomic understanding of this genus remains limited, as previous studies have relied exclusively on morphological characteristics, and molecular phylogenetic investigations are notably lacking. In this study, we propose a new species of the genus *Epipyxis* from Shanxi Province, China. This species exhibits a colonial lifestyle, with cells reproducing via longitudinal division, where the loricae of young individuals attach to the inner surface of that of older individuals. Through detailed observation, we found that the bending and swaying of the longer flagellum are closely associated with cell motility, suggesting that the periodic oscillation of the longer flagellum plays a crucial role in driving cell movement.

The identification of the genus *Epipyxis* is based on structural characteristics such as lorica morphology, protoplast shape, flagellar length, and the presence of red stigmas. Additionally, the surface of the loricae is decorated by scales, which vary in shape, including circular, elliptical, and ovate forms (Petersen and Hansen 1958; Hilliard and Asmund 1963). In our study, the margins of loricae were smooth and nearly parallel, the surface of loricae was adorned with ovate or elliptical scales. The protoplast was spindle, and the two flagella exhibited a length ratio of 1:3, all of which serve as diagnostic features for species identification. The stigma, typically located atop the chromatophore or dorsally appended to it, is another critical taxonomic characteristic of this genus (Bourrelly 1957). Skuja (1956) proposed that stigmas may disappear as the protoplasts age, however, our observations revealed that each cell contained a single protoplast with a stigma positioned at the apical center. Notably, we encountered a rare instance of cells with two stigmas, which may indicate that the cells were undergoing or preparing for division. Based on these findings, we contend that the presence of a stigma cannot be used as an indicator of protoplast age. Furthermore, phenotypic plasticity adds complexity to species identification within this genus, posing significant challenges for accurate taxonomic classification.

*Epipyxisfenheensis* sp. nov. is a unicellular organism characterized by elongate tubular loricae. The lorica features a slightly expanded or unexpanded upper opening and parallel lateral margins. Its surface is ornamented with ovate or elliptical scales. The protoplast is spindle-shaped, tapering posteriorly into a stalk. The long flagellum is equal in length to the protoplast, whereas the short flagellum measures approximately one-third of the protoplast length. We conducted a detailed morphological comparison between *E.fenheensis* sp. nov. and other known *Epipyxis* species (Suppl. material 5). While *E.ramosa*, *E.pulchra*, and *E.elegans* also possess long cylindrical loricae, they are distinguished by their conspicuously dentate surface ornamentation. Different staining methods may influence the observed morphology of the loricae. In *E.michiganensis*, methylene blue staining revealed serrated surfaces whereas Jensen staining yielded smooth morphologies (Hilliard 1964). In our study, the surface of the loricae in *E.fenheensis* sp. nov. was smooth after staining by both methods. Similarly, species with smooth lorica surfaces exhibit clear morphological differences from the new species. *E.thamnoides* shares a similar lorica shape with *E.fenheensis* sp. nov., but its size is much larger. In contrast, *E.epiplanctica* and *E.deformans* possess shorter loricae, with the latter further differentiated by its funnel-shaped opening and conical posterior apex—features inconsistent with the sparallel lateral margins of *E.fenheensis* sp. nov. The loricae and protoplasts of *E.kenaiensis* are smaller than those of *E.fenheensis* sp. nov., and its lorica exhibits an enlarged posterior region with an incompletely smooth lateral margin. While *E.socialis* and *E.stokesii* display lorica shapes resembling the new species, they are distinguished by their longer dimensions and lack of detailed descriptions of their protoplasts, scales, and flagella. *E.irregularis* differs markedly from *E.fenheensis* sp. nov. in its greater lorica length, straight to semi-curved lateral margins, and irregularly parallel surface.

The application of molecular markers has become a well-established method for distinguishing protist species and assessing their diversity (Krienitz and Bock 2012; Leliaert et al. 2014). Early molecular studies primarily relied on small subunit (SSU) ribosomal RNA gene data. The pioneering work by Andersen et al. (1999) illuminated distinct clades of uniflagellate and biflagellate organisms, inferring a weak relationship between *E.aurea* and *E.pulchra* based on SSU rRNA phylogeny. However, due to its high degree of conservation, the SSU rRNA gene may not fully capture genetic diversity or resolve complex phylogenetic relationships within closely related lineages (Bock et al. 2010; Nassonova et al. 2010; Škaloud et al. 2012). Subsequent studies incorporated the *rbc*L gene, with sequences determined for *E.aurea* and *E.pulchra* (Daugbjerg and Andersen 1997; Andersen 2007). More recently, Pusztai and Škaloud (2019) conducted a phylogeny of Chrysophyceae based on concatenated SSU rDNA and *rbc*L dataset, strongly supporting a close relationship between *E.aurea* and *E.pulchra* with high supporting values. While these studies have expanded the available primers for this genus, the broader applicability of these primers across protist taxa remains uncertain. LSU has also proven valuable in phylogenetic studies, offering resolution comparable to SSU while exhibiting higher genetic divergence (Bock et al. 2017). In this study, we conducted phylogenetic trees based on concatenated SSU, LSU, and *rbc*L sequences. Our results further corroborate the close relationship between *E.aurea* and *E.pulchra*, and we suggest proposing a new species *E.fenheensis* sp. nov. based on molecular evidence. In addition, the newly proposed classification system (Guiry 2024) breaks the previous conclusion that Ochromonadales, Hibberdiales, Hydrurales, Chromulinales, and Paraphysomonadales represent monophyletic orders based on recent phylogenetic reconstructions. Given the need for further validation, this study adheres to the previous classification system. We emphasize the importance of molecular sequencing in refining taxonomic frameworks and argue that new classification systems require more molecular support. Expanding molecular datasets will enhance our understanding of interspecific relationships within the genus *Epipyxis* and reveal the species diversity of Chrysophyceae. In addition, we recommend an integrated approach combining morphological and molecular data for taxonomic assessments of freshwater Chrysophyta, as this provides a more robust understanding of both species diversity and geographic distribution.

Fossil calibrations have been successfully applied to the evolutionary study of chrysophytes (Jo et al. 2013; Siver et al. 2015; Čertnerová et al. 2019). However, the origin and evolutionary history of *Epipyxis* remain largely unexplored. Early studies on diatom ancestry suggested a close relationship with chrysophytes (Pascher 1921; Korsikov 1930), but *Epipyxis* received little attention until Čertnerová et al. (2019) placed *E.aurea* in a time-calibrated phylogeny, revealing the divergence time of *E.aurea* from *Dinobryoncylindricum* during the late Cretaceous period. However, the evolutionary origins of the genus *Epipyxis* remain poorly understood due to limited molecular data. In our study, we supplemented molecular sequences of the genus *Epipyxis* and estimated the divergence time of Chrysophyceae species. Molecular phylogeny will help to discover more new species and trace origin and evolutionary trajectories. Therefore, obtaining more molecular sequences of species will significantly enhance our understanding of species diversity and the evolutionary history of Chrysophyta.

## ﻿Conclusions

In this study, we investigated the diversity of the genus *Epipyxis* through integrated morphological and molecular phylogenetic analyses. Based on unique morphological characteristics and independent phylogenetic position, we proposed a new species, *Epipyxisfenheensis* sp. nov., from China. Our molecular phylogenetic analyses, incorporating newly generated sequence data, provide important insights into the relationships and evolutionary history of this genus. It is inferred that the genus *Epipyxis* likely originated in the Early Cretaceous, and three species *E.aurea*, *E.pulchra*, and *E.fenheensis* sp. nov. most likely diverged between the Eocene Paleogene and the Early Cretaceous. This study enhances our understanding of *Epipyxis* diversity and has the potential guiding significance for further taxonomic revisions within Chrysophyta.

## Supplementary Material

XML Treatment for Epipyxis fenheensis

## ﻿Additional information

### Use of AI

No use of AI was reported.

### Author ORCIDs

Jun-Xue Hao https://orcid.org/0000-0002-7910-5811

Ya-Lu An https://orcid.org/0009-0005-5732-1950

Fang-Ru Nan https://orcid.org/0000-0002-4490-4912

Jun-Ping Lv https://orcid.org/0000-0003-1320-7070

Qi Liu https://orcid.org/0000-0002-8710-4560

Xu-Dong Liu https://orcid.org/0000-0003-1679-5584

Shu-Lian Xie https://orcid.org/0000-0003-2349-2071

Jia Feng https://orcid.org/0000-0003-2132-2503

### Data availability

All of the data that support the findings of this study are available in the main text or Supplementary Information.

## Supplementary materials

Supplementary material 1 Taxa and accession numbers used in this study. Newly acquired strain is highlighted in bold Data type xlsx https://binary.pensoft.net/file/1383336This dataset is made available under the Open Database License (http://opendatacommons.org/licenses/odbl/1.0/). The Open Database License (ODbL) is a license agreement intended to allow users to freely share, modify, and use this Dataset while maintaining this same freedom for others, provided that the original source and author(s) are credited.Jun-Xue Hao

Supplementary material 2 Pairwise distance (lower-left matrix) and number of nucleotide variance (upper-right matrix) of SSU sequence among the taxa in this study Data type xls https://binary.pensoft.net/file/1383341This dataset is made available under the Open Database License (http://opendatacommons.org/licenses/odbl/1.0/). The Open Database License (ODbL) is a license agreement intended to allow users to freely share, modify, and use this Dataset while maintaining this same freedom for others, provided that the original source and author(s) are credited.Jun-Xue Hao

Supplementary material 3 Pairwise distance (lower-left matrix) and number of nucleotide variance (upper-right matrix) of LSU sequence among the taxa in this study Data type xls https://binary.pensoft.net/file/1383342This dataset is made available under the Open Database License (http://opendatacommons.org/licenses/odbl/1.0/). The Open Database License (ODbL) is a license agreement intended to allow users to freely share, modify, and use this Dataset while maintaining this same freedom for others, provided that the original source and author(s) are credited.Jun-Xue Hao

Supplementary material 4 Pairwise distance (lower-left matrix) and number of nucleotide variance (upper-right matrix) of rbcL sequence among the taxa in this study Data type xls https://binary.pensoft.net/file/1383343This dataset is made available under the Open Database License (http://opendatacommons.org/licenses/odbl/1.0/). The Open Database License (ODbL) is a license agreement intended to allow users to freely share, modify, and use this Dataset while maintaining this same freedom for others, provided that the original source and author(s) are credited.Jun-Xue Hao

Supplementary material 5 Summary of the features that distinguish apart the *Epipyxis* taxa Data type xlsx https://binary.pensoft.net/file/1383337This dataset is made available under the Open Database License (http://opendatacommons.org/licenses/odbl/1.0/). The Open Database License (ODbL) is a license agreement intended to allow users to freely share, modify, and use this Dataset while maintaining this same freedom for others, provided that the original source and author(s) are credited.Jun-Xue Hao
